# Severe inbreeding depression in an ecologically important grass is revealed by examining germination, not seed production

**DOI:** 10.1093/aobpla/plag012

**Published:** 2026-02-19

**Authors:** Raelene M Crandall, Carolina Baruzzi, Jennifer M Fill, Opeyemi A Adedoja

**Affiliations:** School of Forest, Fisheries, and Geomatics Sciences, Institute of Food and Agricultural Sciences, University of Florida, P.O. Box 110410, Gainesville, FL 32611, United States; School of Forest, Fisheries, and Geomatics Sciences, Institute of Food and Agricultural Sciences, University of Florida, P.O. Box 110410, Gainesville, FL 32611, United States; Department of Wildlife Ecology and Conservation, North Florida Research and Education Center, Institute of Food and Agricultural Sciences, University of Florida, 155 Research Rd, Quincy, FL 32351, United States; School of Forest, Fisheries, and Geomatics Sciences, Institute of Food and Agricultural Sciences, University of Florida, P.O. Box 110410, Gainesville, FL 32611, United States; Department of Entomology and Nematology, Institute of Food and Agricultural Sciences, University of Florida, 1881 Natural Area Drive, Steinmetz Hall, Gainesville, FL 32611, United States; Department of Biology, University of Central Arkansas, 201 Donaghey Ave, Conway, AR 72035, United States

**Keywords:** *Aristida beyrichiana*, supplemental pollen, pollen identity, pollen availability, pollen compatibility, plant reproduction, wind pollination, wiregrass, restoration

## Abstract

Pollen availability and self-compatibility studies in grasses inform restoration by identifying limitations to germinable seed production. Seed set alone indicates pollen limitation and self-fertilization ability but not self-compatibility, as seeds may not germinate. We tested whether wiregrass (*Aristida beyrichiana*) is pollen-limited and self-compatible in two southeastern US pine savannas differing in ecology and fire frequency. Pollen limitation and self-compatibility were assessed by comparing seed set and germination from naturally pollinated inflorescences, those supplemented with outcross pollen, and those restricted to self-pollen. All treatments produced seeds, but filled and germinable seed numbers varied by site. The wetter, triennially burned site produced significantly more germinable seeds than the drier, annually burned site. Pollen was not limiting: open and outcross-supplemented inflorescences produced similar seed numbers within each site. Based on seed production, wiregrass appeared capable of self-fertilization, but germination data revealed near-zero viability of selfed seeds, indicating severe inbreeding depression rather than true self-incompatibility. Thus, wiregrass is not pollen-limited but exhibits strong embryonic inbreeding depression, implying an obligate outcrossing mating system. This helps explain why wiregrass often produces few viable seeds despite high seed production. Our findings highlight the importance of including germination data in reproductive studies, as seed production alone can mask other biological constraints. For wiregrass, frequent fires likely promote pollen movement and successful outcrossing. These insights inform restoration efforts by clarifying reproductive limitations in a foundational pine savanna species. This has important implications for restoration efforts, particularly in sourcing and evaluating seed material for ecological resilience and long-term establishment.

## Teaser

Wiregrass produces abundant seeds from self-pollination, but germination tests revealed these seeds almost never sprout. This severe inbreeding depression was invisible when counting seeds alone, demonstrating a critical flaw in how plant reproduction is assessed. For effective restoration and accurate pollination studies, seed quality testing is essential. Our findings show that what appears viable may not be, with important implications for understanding plant mating systems and improving restoration outcomes.

## Introduction

The relationship between ovule production and seed set remains a topic of debate in ecology. For a plant to produce a germinable seed, the flower must first receive pollen, either from flowers on the same plant or from those on other plants. Few ovules will be fertilized and/or have the potential to develop into germinable seeds if pollen sources or pollinators are limited and plants are unable to self-pollinate. Second, the pollination process must result in successful fertilization. In self-incompatible species, pollen from genetically similar individuals is commonly rejected before fertilization (Silva and Goring 2001). Depending on the species, however, self-incompatible pollen can be rejected at any time during the pollination process, even after fertilization (Yang et al. 2008). In self-compatible species, pollen from either source can successfully fertilize the ovule, and a germinable seed should develop. In predominantly outcrossing species with a low index of plant fitness, however, inbreeding depression, such as through embryo abortion after fertilization, can prevent the development of a fully germinable seed despite self-fertilization (Guillaume and Jacquemart 1999). Pollen availability and the index of plant fitness of a species can therefore largely determine the reproductive outcome of plant populations (e.g. Knight et al. 2005, Ward and Johnson 2005, Koski et al. 2018, Schermer et al. 2019). Understanding the factors that affect the production of germinable seeds is crucial for understanding fluctuations in plant population dynamics and for developing effective methods for habitat restoration and management (Buisson et al. 2021, Tevlin et al. 2025).

Researchers employ standard methods to determine the roles of pollen availability and self-compatibility in seed production (Knight et al. 2005, Adedoja et al. 2021, Biella et al. 2021). Pollen limitation (i.e. inadequate pollen availability) and index of plant fitness are determined after quantifying differences between seed set in open, naturally pollinated flowers versus pollen-supplemented or bagged flowers, respectively (Harmon-Threatt et al. 2009, Geerts and Adedoja 2021). These treatments are often applied to different flowers or inflorescences on the same individual so that seed set is not confounded by maternal effects (Hamilton and Mitchell-Olds 1994, Biella et al. 2021). However, estimates of pollen limitation can be inflated when treatments are applied to different individuals (Knight et al. 2006).

Seed production does not always reflect pollination success or seed viability. While seed production is commonly used to assess pollination success, it does not always reflect seed viability or potential for germination. Seed production (i.e. number of seeds produced or seed set) is typically considered an indicator of pollination success (Knight et al. 2005, Baskin and Baskin 2015). The number of seeds produced, however, may not always correlate with the number of seeds that can germinate (Aizen and Harder 2007, Alonso et al. 2013). The production of seeds, therefore, does not necessarily indicate complete self-compatibility, as seed abortion can occur even after fertilization (Yang et al. 2008). For example, Geerts and Adedoja (2021) found differences in the number of fruits produced but not in the number of seeds germinated when comparing naturally and self-pollinated *Lythrum salicaria* flowers. Additionally, Hamilton and Mitchell-Olds (1994) observed no significant difference in the proportion of seeds that failed to germinate among pollination treatments. However, seed weight varied among maternal plants across pollination treatments. Considering only the formation of seeds and not their germination could hamper conservation and restoration efforts, especially if most seeds are not viable (Baskin and Baskin 2015, Frischie et al. 2020).

Self-incompatible species predominate among the Poaceae (Yang et al. 2008), a family with many species of high economic value. Understanding seed ecology, from production to germination, of plant species with high ecological and economic value can significantly increase our capacity to sustain agricultural systems and restore natural ecosystems (Buisson et al. 2021). Wiregrass (*Aristida beyrichiana* Trin. & Rupr.) is a perennial bunchgrass often targeted for restoring pine savannas in the southeastern USA (Noss 1989, Peet 1993, Glitzenstein et al. 1995). Although this grass typically produces many seeds with low germination rates (Outcalt 1994, Pérez 2014, Baruzzi et al. 2022, Fill et al. 2021), it has been shown to quickly establish on degraded sites (Mulligan et al. 2002, Trusty and Ober 2009, Love et al. 2024). We tested wiregrass for self-compatibility with pollen exclusion bags and for pollen limitation with outcrossed pollen supplementation to determine if the low germination rate might be associated with the index of plant fitness or with pollen limitation. This study repeats one by Burt and Brudvig (2019), who found insufficient evidence for self-compatibility. We questioned whether this result was due to their chosen response variable (i.e. the number of seeds produced). Thus, we used both the number of seeds produced and the number of seeds germinated as response variables in our experiment. In addition, we conducted this study in two sites that varied in ecological features and fire frequency. Given the importance of fire in this ecosystem, and because fire frequency can affect resource availability and wiregrass reproductive investment (e.g. Fill and Crandall 2024), we also examined whether site-level differences in fire regime influenced seed production and germination outcomes.

## Materials and methods

### Site description

This study was conducted in the >840-hectare Austin Cary Forest, a University of Florida teaching and research forest located northeast of Gainesville, Florida, USA (29.7524° N, 82.2184° W). The climate is humid and subtropical, with an average annual precipitation of 127 cm, primarily occurring during the wet season (late May to early October). The landscape includes large tracts of mesic pine savannas, with some xeric pine savannas, hardwood hammocks, lowland cypress depressions, and small, isolated wetlands. The mesic pine savannas used in our experiment were dominated by *Pinus palustris* (longleaf pine), with occasional *Pinus elliottii* (slash pine) and *Quercus laevis* (turkey oak) in the overstory. In the understory, wiregrass individuals co-occurred with *Carphephorus* sp. (chaffhead), *Liatris spicata* (dense blazing star), *Pityopsis graminifolia* (silkgrass), *Chamaecrista nictitans* (sensitive partridge pea), *Dichanthelium* spp. (panicgrasses), and several other grass species. The density of shrubs, including *Serenoa repens* (saw palmetto), *Ilex glabra* (gallberry), *Gaylussacia dumosa* (dwarf huckleberry), *Quercus minima* (dwarf live oak), and *Vaccinium myrsinites* (blueberry), varied across the landscape.

We selected wiregrass individuals in two pine savanna sites that differed in past fire frequency and ecological features. Henceforth, these sites will be referred to as the annual and triennial burn sites, but they have several differences beyond fire frequency. The annual (3.6 hectares) and triennial (5.3 hectares) burn sites have had prescribed fires applied at 1- and 3-year intervals, respectively, since 1979 and 2012. The sites were burned during the year of this study, 16 and 25 June 2020, during the growing season after the yearly wet season had commenced (Fill et al. 2019). This burn timing is optimal for enhancing the wiregrass flowering response and increasing the production of viable seeds (Outcalt 1994, Fill et al. 2012, Baruzzi et al., 2022). Plant communities were similar at both sites (i.e. wiregrass co-occurred with similar species), and we observed that the understory had less vegetative cover at the annual burn site than at the triennial burn site. The annual burn site also had a lower basal area of overstory trees with an average of 32.8 m^2^/hectare compared to the triennial burn site with a mean of 21.1 m^2^/hectare. The annual burn site was at a slightly higher elevation (43.9 m) with very deep, poorly drained Sparr fine sand, whereas the triennial burn site (43.2 m) had poorly drained Lochloosa fine sand (Thomas et al. 1985). Both were on 0%–8% slopes.

### Study species

Wiregrass, a perennial C4 bunchgrass, is a widespread component of pine ecosystems in the southeastern Coastal Plain. In the absence of fire, wiregrass plants primarily spread vegetatively by producing compact tillers, resulting in an increasingly larger plant that eventually fragments into ostensibly separate plants (Clewell 1989, Laucevicius et al. 2021, Fill and Crandall 2024). It reproduces after fires but is sensitive to burn season (Parrott 1967, Clewell 1989, Fill et al. 2016), with most prolific flowering after fires that occur soon after the onset of the summer wet season (late May through June) (Outcalt 1994, Fill et al. 2012, Baruzzi et al. 2022). Fire season has been shown to interact with microsite conditions; wiregrass plants may produce more seeds after being burned during the early wet season if they are under partial canopy cover (Baruzzi et al. 2022). Pollination of wiregrass’s tightly packed flowers is thought to be primarily by wind (Burt and Brudvig 2019). Still, ants and other insects have been observed on the flowers and likely contribute to pollen transfer between plants.

### Study design and data collection

To investigate pollen limitation and the index of plant fitness in wiregrass, we randomly selected and tagged 20 individual plants spread over an area of approximately 0.2 km^2^ in both the annual and triennial burn sites (40 plants in total). Individuals were at least 5 m apart to increase the likelihood that each was a distinct genet, although we acknowledge that this distance may not guarantee genetic distinctness, given that wiregrass can spread vegetatively (Clewell 1989, Fill et al. 2026). On each plant, all flowers on three randomly selected inflorescences received one of three treatments: (i) open-pollinated were exposed to pollen transported by the wind or pollinators without manipulation; (ii) outcross-pollen supplementation described below; (iii) autonomous self-fertilization, in which inflorescences were covered with pollination bags before having receptive stigmas to prevent outcrossed pollen from coming in contact with floral structures via wind or pollinators. Self-fertilized inflorescences were covered with natural kraft bags while receptive to pollen.

Outcross-pollen supplementation began and ended with anther emergence and dehiscence, respectively, on each inflorescence. Flowers opened from the base of the inflorescence towards the tip, necessitating multiple treatments beginning with the lower flowers. To pollinate inflorescences, supplemental pollen was gathered with paintbrushes by sweeping the anthers of ten wiregrass individuals at least 10 m from study plants before applying it to the stigmas of flowers on outcross-pollen supplemented inflorescences. In total, outcross-pollen supplemented inflorescences were treated every 1–2 days for up to six visits over the duration of the flowering period (30 September to 19 October 2020).

Study plants were monitored weekly for seed maturation. Seeds matured in different weeks within and between plants at the annual site, but all seeds matured at the same time at the triennial site. Thus, to prevent granivory at the annual site, all treated inflorescences in that unit were covered with woven polypropylene bags after they ceased flowering. Once seeds were visibly mature, we collected entire inflorescences and counted the number of seeds by pollination treatment and plant at both sites. Seeds were then cold stratified (4°C) for approximately eight weeks before initiating germination trials (Fill et al. 2021, 2024).

We tested for differences in germination by site and pollination treatment among plants with undamaged inflorescences (i.e. those that did not appear broken; Annual: *n* = 19; Triennial: *n* = 18). Although we initially tagged 40 plants (20 per site) with three inflorescences each (one per treatment), some inflorescences were bent or detached before seed maturation due to herbivory (e.g. grasshoppers) and trampling (e.g. deer). We excluded these plants (i.e. 1 in the annual and 2 in the triennial burn sites) because we could not determine whether the absence of seeds was a biological result or an artefact of physical damage. This also ensured we had only plants with values for all three treatments. We placed all seeds from each treated inflorescence, along with their attached lemmas and paleas, in separate germination boxes on blotter paper and added 4 ml of a 0.002% solution of Plant Preservative Mixture (Plant Cell Technology, Washington, DC, USA) to reduce fungal contamination. The containers were left at room temperature (21–23°C) with no direct sunlight for approximately 2.5 months. Germination boxes were randomly arranged on the bench and repositioned weekly to minimize positional effects (Fill et al. 2021, Baruzzi et al. 2022). The seeds were monitored every three days for signs of germination, denoted by epicotyl emergence. As seeds germinated, they were recorded and removed from boxes.

### Statistical analysis

We used non-parametric bootstrapping with 95% confidence intervals to visualize the differences between each pair of experimental treatments at each site. To determine significant differences between treatments and sites for the number of seeds produced and seeds germinated per inflorescence, 95% confidence intervals of mean pairwise differences were calculated (Table 1). If the 95% confidence interval of the difference contained zero, there was no significant difference between the treatment outcomes (Efron and Tibshirani 1994).

**Table 1:** Pairwise comparisons from the mean of the bootstrapped distribution for the number of wiregrass seeds produced (a) and seeds germinated (b) per inflorescence at each burn site (annual or triennial burn frequency) and pollination treatment (autonomous-, open-, or outcross-pollinated).

	Annual, autonomous	Annual, open	Triennial, outcrossed	Triennial, autonomous	Triennial, open (44.2 ± 21.4)
a. *Number of seeds produced per inflorescence*					
Annual, outcrossed (30.7 ± 20.0)	−10.1, 9.6*	−11.1, 12. 7*	−11.6, 12.6*	−24.8, −0.02	−26.5, −0.1
Annual, autonomous (32.1 ± 10.6)		−7.5, 10.7*	−8.5, 10.0*	−21.1, −1.1	−23.2, −2.2
Annual, open (30.2 ± 19.10)			−11.5, 11.1*	−24.0, −1.1	−26.7, −1.9
Triennial, outcrossed (31.0 ± 17.9)				−23.2, −0.6	−24.9, −1.0
Triennial, autonomous (43.3 ± 18.4)					−14.3, 11.6*
b. *Number of seeds germinated per inflorescence*					
Annual, outcrossed	0.05, 1.8	−1.3, 1.36*	−5.7, −0.7	0.00, 1.7*	−8.0, −1.6
Annual, autonomous		−1.8, 0.05*	−6.3, −1.7	−0.3, 0.1*	−8.7, −2.5
Annual, open			−5.7, −0.8	−0.1, 1.7*	−8.1, −1.4
Triennial, outcrossed				1.7, 6.2	−5.6, 2.3*
Triennial, autonomous					−8.6, −2.3

Asterisks indicate a significant difference between the comparisons (i.e. interval contains zero). In (a), we also indicate the average number of seeds per individual ± standard deviation below each treatment.

The index of plant fitness and pollen limitation was calculated using two measurement types: the number of seeds produced and the number of seeds germinated. The index of plant fitness was calculated as the ratio of results in the bagged versus outcross-pollen supplemented treatments (Harmon-Threatt et al. 2009). Pollen limitation was calculated as:


(1) $$\mathrm{Pollen}\;\mathrm{limitation}\;=\;\frac{\ln(\mathrm{open}\;\mathrm{pollinated}+1)}{\ln(\mathrm{outcrossed}\;\mathrm{pollinated}+1)}$$

Individual plants were considered the unit of replication; thus, the index of plant fitness and pollen limitation were calculated using results per plant individual at the inflorescence level. When the index of plant fitness or pollen limitation did not differ from zero (i.e. 95% confidence intervals overlapped zero), there was no evidence of the phenomenon.

We used two complementary statistical approaches to account for modest sample sizes and the risk of Type II error. First, we employed non-parametric bootstrapping with 95% confidence intervals to visualize differences between treatments at each site, calculating mean pairwise differences as described above. We chose non-parametric bootstrapping because it is well-suited for small, unbalanced sample sizes and does not require the distributional assumptions of parametric models (Efron and Tibshirani 1994). Second, we used generalized linear mixed models (GLMMs) and generalized linear models (GLMs) to test for treatment effects across both sites. For seed production counts, we fitted a negative binomial GLM using the MASS package (Venables and Ripley 2002) to account for overdispersion, as a Poisson model showed poor fit (residual deviance = 1073.0 on 108 df). For germination data, we fitted a binomial GLMM with plant identity as a random effect using the lme4 package (Bates et al. 2015) to account for multiple seeds per plant. Pairwise treatment comparisons were conducted using estimated marginal means with Tukey adjustment for multiple comparisons using the emmeans package (Lenth 2024). All analyses were conducted in R version 4.5 (R Core Team 2025).

## Results

**Figure 1:**
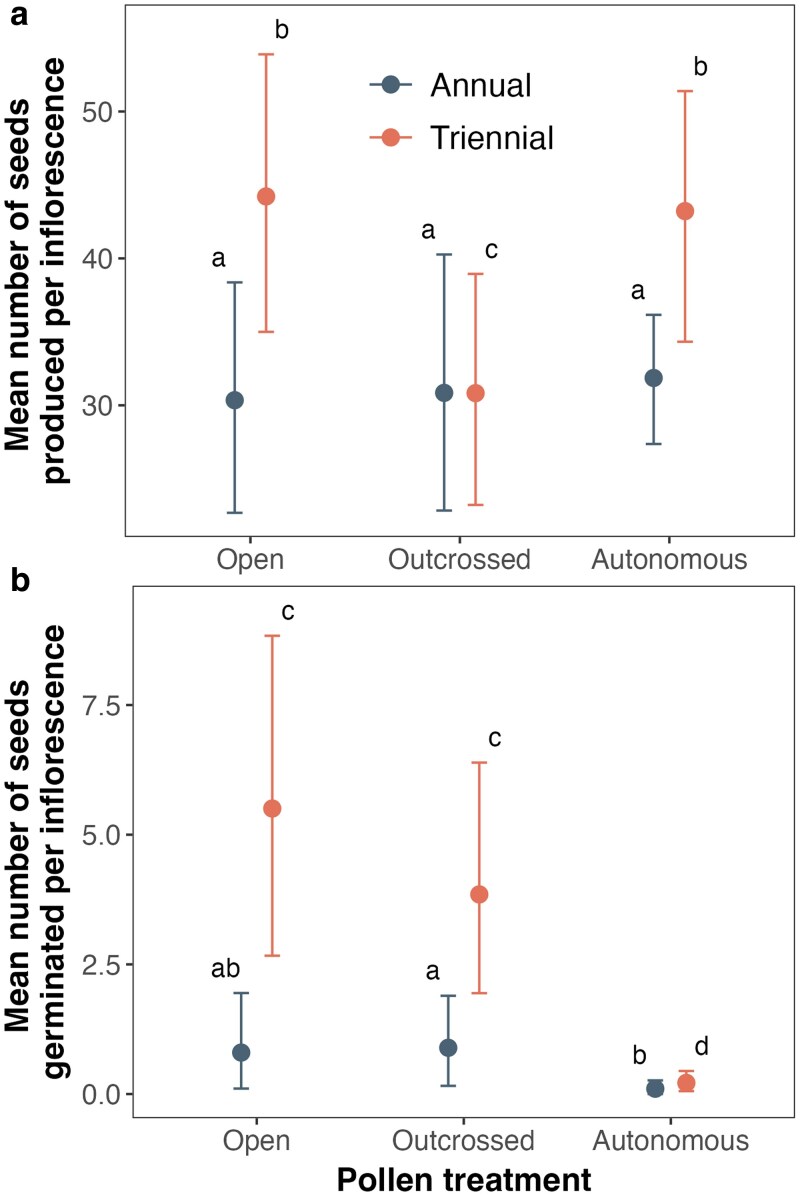
The bootstrapped mean and 95% confidence intervals for the number of wiregrass (*Aristida beyrichiana*) seeds produced (a) and seeds germinated (b) per inflorescence at each burn site (annual vs. triennial burn frequency) and pollination treatment (open, outcrossed, or autonomous). Means with different letters are significantly different based on 95% bootstrapped confidence intervals. Also see Table 1a and b for significance values.

All wiregrass plants formed seeds on treated inflorescences, ranging from 1 to 97 seeds, with an average of 35.1 seeds per inflorescence (Table 1). The mean number of seeds produced per inflorescence differed by treatment and site (Table 1a; Fig. 1a). The number of seeds produced per inflorescence was significantly higher at the triennial burn site than at the annual burn site for the autonomous and open treatments. There were no differences between sites for the outcross-pollen supplemented treatment. There were no significant differences between treatments for the number of seeds produced at the annual burn site. In contrast, the autonomous and open inflorescences produced significantly more seeds at the triennial burn site than all treatments at the annual burn site and the outcross-pollen supplemented treatment at the triennial burn site.

The number of seeds germinated also differed significantly among pollination treatment and between sites, but the patterns differed from the number of seeds produced per inflorescence (Table 1b; Fig. 1b). At the annual burn site, there were no differences in the number of seeds germinated between pollination treatments. In contrast, the outcross-pollen supplemented and open flowers at the triennial burn site had more germinable seeds than did the autonomous flowers. There was no significant difference in the number of seeds germinated between the autonomous treatments at the two sites, which were both near zero. For the outcross-pollen supplemented and open treatments, the triennial burn site had significantly higher germination than the annual burn site.

Similarly, seed production did not differ significantly among pollination treatments when analyzed with a negative binomial GLM (all pairwise comparisons *P* > 0.27). In contrast, germination differed significantly among treatments in the binomial GLMM. The autonomous self-fertilization treatment showed significantly lower germination than both the outcross-pollen supplemented treatment (estimate = 3.32 log odds, *P* < 0.0001) and the open-pollinated treatment (estimate = 3.37 log odds, *P* < 0.0001). Germination did not differ significantly between outcross-pollen supplemented and open-pollinated treatments (estimate = 0.044 log odds, *P* = 0.963). These formal hypothesis tests corroborate the bootstrapped confidence interval results (Table 1), confirming that while wiregrass produces seeds from self-pollination, these seeds exhibit near-zero germination success.

**Figure 2:**
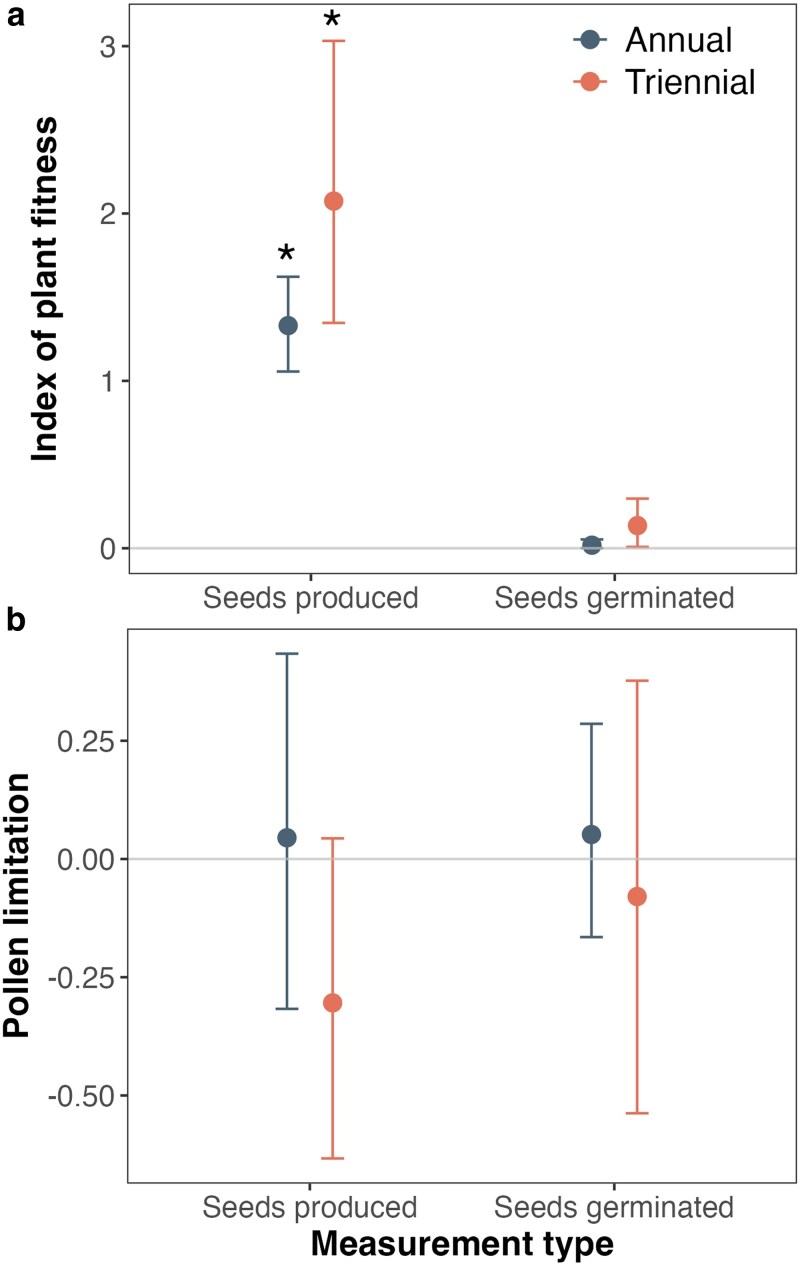
The bootstrapped mean and 95% confidence intervals for calculations of the index of plant fitness (a; ratio of autonomous to outcross-supplemented results) and pollen limitation (b) using wiregrass (*Aristida beyrichiana*) seeds produced or germinated in two sites characterized by annual or triennial burn frequencies. An asterisk above the bar indicates that the 95% confidence interval does not include zero.

The response variable used to detect the index of plant fitness affected our inference. When the number of seeds produced was used to determine the index of plant fitness, it was greater than zero for both sites, suggesting wiregrass can self-fertilize (Fig. 2a). In contrast, the index of plant fitness based on germinated seeds was near zero, indicating severe post-zygotic inbreeding depression rather than pre-zygotic self-incompatibility; selfed embryos form but fail to develop into viable seeds. Furthermore, although the results show high variation, the calculations for pollen limitation at both burn sites and measurement types indicate that pollen is not limited (i.e. it does not differ significantly from zero; Fig. 2b).

## Discussion

**Figure 3:**
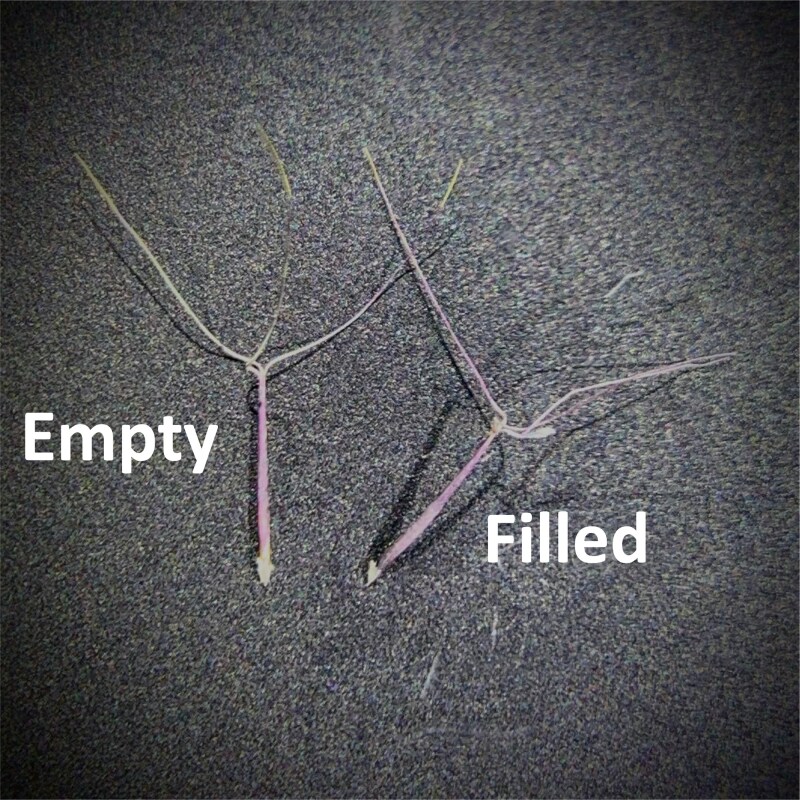
Two wiregrass (*Aristida beyrichiana*) seeds that appear similar but have different likelihoods of germinating. Although we used germination trials in our experiment, it is also possible to use the ‘enhanced forceps press test’. If seeds remain rigid when gently pressed on with forceps, they are likely filled (Pérez and Norcini 2010) and thus have a higher probability of germination (Fill et al. 2021, Baruzzi et al. 2022, Fill and Crandall 2024). Photo credit: R.M. Crandall.

Quantifying seed production alone might lead to erroneous conclusions in plant reproduction studies. In this experiment, we showed that the true reproductive consequences of self-pollination were only evident when quantifying seed germination of wiregrass (*A. beyrichiana*). When the number of germinated seeds was used to assess reproductive success, evidence suggested that wiregrass exhibits severe post-zygotic inbreeding depression, making it a functional obligate outcrosser despite being capable of self-fertilization. In contrast, considering only the number of seeds produced, as done by Burt and Brudvig (2019), led to the conclusion that wiregrass is self-compatible. Incorporating a seed quality test, such as germination trials, tetrazolium tests, or imaging, should increase the accuracy of pollination studies (Frischie et al. 2020). This is particularly important for those species with fruits or seeds that appear similar despite differing quality (Fig. 3; e.g. Geerts and Adedoja 2021).

Environmental conditions influenced germination rates in the outcrossed treatments. Plants in the more mesic, triennially burned site likely had greater access to water than those in the drier, annually burned site, potentially supporting greater production of germinable seeds (Recart et al. 2019). The longer interval since the last fire also led to higher fuel loads, which can enhance post-fire reproductive output (Brewer et al. 2009, Gagnon et al. 2012). In addition, the longer fire interval might also allow for sufficient recovery of plant reserves to allocate to the production of filled seeds (Jose et al. 2006, Bond and van Wilgen 2012). The capacity of seeds to germinate is known to be affected by multiple factors, including water, light, nutrient availability, and soil conditions (Outcalt 1994, Kilkenny and Galloway 2008, Fujita et al. 2014, Recart and Campbell 2021, Baruzzi et al. 2022, Mehmood et al. 2025), pollen quality (Iwaizumi and Takahashi 2012, Rosbakh et al. 2018, Mesnoua et al. 2024), and genetic load from accumulated somatic mutations (Lamont and Wiens 2003). Additionally, wiregrass seeds are often infected with a smut fungus (Fill et al. 2024), which may also be influenced by environmental variables. It is possible that, at the triennial site, more belowground resources were allocated to inflorescences receiving heavier outcross pollen loads, a scenario in which pollen limitation is more likely to be detected at the inflorescence level (Knight et al. 2006). Nonetheless, when we used methods consistent with Burt and Brudvig (2019), our results based on seed counts were similar, reinforcing the role of environmental context.

Pollen movement in wind-pollinated species, such as wiregrass, may be influenced by the structure of their habitat. We did not find evidence of pollen dispersal barriers in our study populations, likely because wiregrass flowers soon after fire in an ecosystem dominated by resprouting plants (Brockway and Lewis 1997, Fill et al. 2012, 2021). When wiregrass is flowering, taller shrubs are still resprouting near the soil surface (Wiesner et al. 2019, Lacouture et al. 2020), reducing vertical obstructions. However, this may not be the case for other *Aristida* species. Unlike wiregrass, some co-occurring *Aristida* species do not flower in response to fire but are also wind-pollinated (Cerros-Tlatilpa et al. 2011, Shepherd et al. 2012). It remains unclear whether these species rely on periodic fire to ensure sufficient pollen dispersal and successful reproduction.

Assessing seed quality is especially important for restoration, where project success often hinges on having seeds that are filled and capable of germinating. Wiregrass seeds can cost up to $1200 per pound, considering the labour-intensive and site-specific nature of seed collection (Carney et al. 2024). Yet, wiregrass is typically the first and most widely planted species when restoring pine savanna understories (Brockway et al. 2005, Aschenbach et al. 2010, Fill et al. 2021, Love et al. 2024). Given its ecological and economic importance, a clear understanding of wiregrass pollination biology and seed quality is essential for effective restoration planning.

One of the most significant bottlenecks to restoring natural areas is acquiring seeds capable of germinating and establishing. We identified three potential limitations to procuring germinable wiregrass seeds: (i) outcrossing is needed; (ii) there are site- or disturbance-level effects that might influence germination capacity; and (iii) all seeds visually appear similar. Wiregrass flowering culms produce numerous seeds that vary little in appearance, but they do differ in their ability to germinate (Pérez and Norcini 2010, Baruzzi et al. 2022, Fill et al. 2021, Tevlin et al. 2025). Germination success from selfed seeds was near zero, demonstrating severe inbreeding depression rather than true self-incompatibility; wiregrass can self-fertilize, but selfed offspring rarely survive. As Henry David Thoreau (1993) wrote, ‘I have great faith in a seed… Convince me that you have a seed there, and I am prepared to expect wonders’. Likewise, studies of pollen limitation and self-compatibility that include seed germination capacity should make a great difference for restoration and management.

## Data Availability

The data are available at Zenodo: https://zenodo.org/records/17063843
